# MEANING FOCUSED COPING: IMPLICATIONS FOR FAMILY CAREGIVER BURDEN AND POSITIVE ASPECTS OF CARE

**DOI:** 10.1093/geroni/igac059.2551

**Published:** 2022-12-20

**Authors:** JoAnna Dieker, Sara Qualls

**Affiliations:** University of Colorado Colorado Springs, San Antonio, Texas, United States; University of Colorado Colorado Springs, Colorado Springs, Colorado, United States

## Abstract

The Stress Process Model (Pearlin et al., 1991) posits that different styles of coping influence both positive and negative psychological outcomes in family caregivers. Meaning-focused coping is hypothesized to promote positive psychological experiences, but presently it is unclear how meaning-focused coping is related to caregiver burden. The present study tested the associations among meaning-focused coping, indicators of burden, and positive aspects of care in adult child and spousal caregivers of older adults with dementia. An online sample of caregivers ranging in age from 30-68 (n = 219) completed a measure of meaning-focused coping (COPE-PRG; Carver et al., 1989) and the Caregiver Reaction Scale (CRS; O’Malley & Qualls, 2016) which assessed overload, role captivity, personal growth, and competence. Regression analyses indicated that meaning-focused coping predicted less role captivity and predicted greater personal growth and competence. However, meaning-focused coping did not account for significant variance in perceived overload. The findings extend prior research by demonstrating that meaning-focused coping strategies may faciliate positive aspects of care during chronically stressful experiences such as caregiving. Findings highlight that meaning-focused strategies may be more helpful for coping with role captivity (e.g., feeling unable to participate in work or family roles outside of caregiving) and less helpful for coping with subjective overload (e.g., a sense of overwhelm) in caregivers. Results inform clinical interventions that promote meaning and reduce burden in caregivers.

